# HTLV-1 infection modulates the immune response in HIV-HCV coinfected patients and may increase spontaneous HCV clearance

**DOI:** 10.1186/1742-4690-11-S1-O8

**Published:** 2014-01-07

**Authors:** Fabianna Bahia, Marcos H Abrahão, Eduardo M Netto, Patricia Bozza, Carlos Brites

**Affiliations:** 1 Laboratório de Pesquisa em Infectologia, Complexo Hospitalar Prof. Edgard Santos – Federal University of Bahia, Brazil; 2 FIOCRUZ , Rio de Janeiro, Brazil

## Background

HTLV-1 often infect HIV-HCV coinfected patients. The immunological consequences of triple infection are still unclear. Objective: to compare the plasma levels of 25 different cytokines in patients infected by HIV and HCV, according to their serological status to HTLV-1 infection.

## Methods

We used the LUMINEX 100/200 analyzer to compare the plasma levels of 25 different cytokines for patients coinfected by HCV and HIV (N=15), or by HTLV-1, HCV and HIV(N=18, 3 of them spontaneously cleared HCV).

## Results

We detected a significant increase in median plasma levels of 6 different cytokines (Table 1) for patients coinfected by HTLV-1.Only the 3 patients with HCV spontaneous clearance showed detectable levels of IL-15 (p=0.06). Levels of remaining 18 cytokines were similar for all groups.

**Table 1:** 

	Median Plasma levels of cytokines, according to type of infection			
		
Cytokines	HIV+ HTLV (spontaneous clearance of HCV)	HIV+ HTLV+HCV	HIV+HCV	P value
		
	N = 3	N = 14	N = 15	
**IL-1b**	88,7	16,6	1,1	**0,01**

**IL-2**	280,4	72,7	15,1	**0,01**

**FGF**	57,8	26,3	14,3	**0,03**

**IFN-g**	1580,7	571,4	224,7	**0,04**

**IP-10**	70896,1	46985,6	3753,9	**0,03**

**MIP-1a**	15862,6	103,0	20,5	**0,00**

**MIP-1b**	14301,3	4732,3	666,5	**0,01**

**TNF-a**	1144,8	155,7	33,7	**0,01**

## Conclusions

Infection by HTLV-1, in HIV-HCV coinfected patients, seems to induce a greater release of pro-inflamatory cytokines. This can be associated with a higher rate of HCV spontaneous clearance.

